# Rewriting destiny—gene-hacked stem cells ignite a revolution against aging

**DOI:** 10.1038/s41419-025-07958-6

**Published:** 2025-08-21

**Authors:** Liangyu Lin, Qun Xue, Gerry Melino, Yufang Shi

**Affiliations:** 1 Shanghai Institute of Nutrition and Health, University of Chinese Academy of Sciences, Chinese Academy of Sciences, Shanghai, China; 2 Department of Neurology, the First Affiliated Hospital of Soochow University, Soochow University, Suzhou, China; 3 Department of Experimental Medicine, TOR, University of Rome Tor Vergata, Rome, Italy; 4 The Fourth Affiliated Hospital of Soochow University, Institutes for Translational Medicine, Suzhou Medical College, Soochow University, Suzhou, China

**Keywords:** Ageing, Senescence

Immortality, hence aging, has always been at the center of human thinking. In the “books”, Methuselah died at 969 years of age. According to Genesis, this central figure of all three monotheistic religions (Judaism, Christianity, Islam) was the son of Enoch, the father of Lamech, and the grandfather of Noah. In fact, Noah’s son lived nearly 500 years, whilst Abraham died at 175. Further, Adam lived 930 years, Seth 912, Enos 905, Kenan 910, Mahalalel 895, Jared 962, Enoch 365 (did not die, but was taken by God), Lamech 777, and Noah 950 (https://en.wikipedia.org/wiki/Patriarchs_(Bible)#:~:text=before%20the%20Flood). So, now the question is, in detail, why do we live so little? Is aging a mechanism for maintaining “quality of life”? Aging is, in fact, humanity’s oldest foe, a silent predator that saps our strength, dulls our minds, and ushers in a cascade of ailments—Alzheimer’s, osteoporosis, infertility, and heart failure, to name a few. It’s a universal truth we have long accepted as inevitable, a one-way ticket to decline. But what if this narrative could be rewritten?

In a landmark study [1] published in the latest issue of Cell (2025;188:1–22), a 44-week trial in aged macaques, led by Guanghui Liu, Si Wang, and Jing Qu, has delivered a scientific paradigm shift: genetically engineered human mesenchymal progenitor(stem) cells, termed senescence-resistant cells (SRCs), can not only halt aging but roll back its clinical and functions negative effects. Delivered intravenously, these supercharged cells sparked a rejuvenation trifecta—sharper cognition, stronger bones, and revitalized reproductive health—all without identifiable adverse effects. The key seems to lie in their microscopic messengers, exosomes, which tackle the root of aging: cellular senescence. But what are the full underlying molecular mechanisms? Are these translatable to humans and how?

The Liu team found that peripheral blood mononuclear cells (PBMCs) exhibited decreased p21^CIP1^ positivity, reduced lipid peroxidation detected by 4-hydroxynonenal, and γH2AX, as well as IL-1β, TNF-α, and IL-6, whilst increased H3K9me3 levels indicated stable heterochromatin. These are signs of active blood functions. Consistently, RNAseq data show distinct clusters related to innate immunity and inflammation. Epigenetic aging (DNAmAge) shows improvement of distinct markers as indicated by increased levels of nuclear lamina component lamin B1 and H3K9me3, enhancing chromatin compaction and gene silencing at lamina-associated domains, contributing to nuclear stability but also promoting nuclear envelope ruptures in aging cells. This leads to Aβ (4G8) and p-Tau (T205) accumulation, activating the cGAS-STING pathway and driving chronic inflammation via immune cells like microglia, PBMCs, and CD45^+^ cells. SRC’s engineered MSC-derived exosomes, enriched with anti-inflammatory miRNAs, longevity proteins, and cytokines, counteract these effects by suppressing inflammation, reducing immune cell infiltration, and promoting tissue rejuvenation. These engineered stem cells offer a promising approach for managing aging-related immune dysregulation and associated chronic diseases.

The study’s implications are of high potential relevance, offering a glimpse into a future where aging is not a descent into frailty but a manageable condition. At the heart of this work are the SRCs, mesenchymal progenitor cells (MPCs) genetically modified to flourish in the harsh environment of an aging body, where chronic inflammation and oxidative stress dominate. Unlike conventional stem cells, SRCs are armed with the geroprotective gene FOXO3, identified by the same team as a powerhouse against aging [2, 3]. This gene activates longevity pathways, fortifying the cells to resist deterioration and combat the ravages of time. In aged cynomolgus macaques, these cells did not just survive—they orchestrated a systemic rollback of aging’s hallmarks. The results seem to be staggering: enhanced brain function, fortified skeletal strength, restored reproductive vigor, and a dramatic reduction in cellular senescence and inflammation (Fig. 1).

**Fig. 1:**
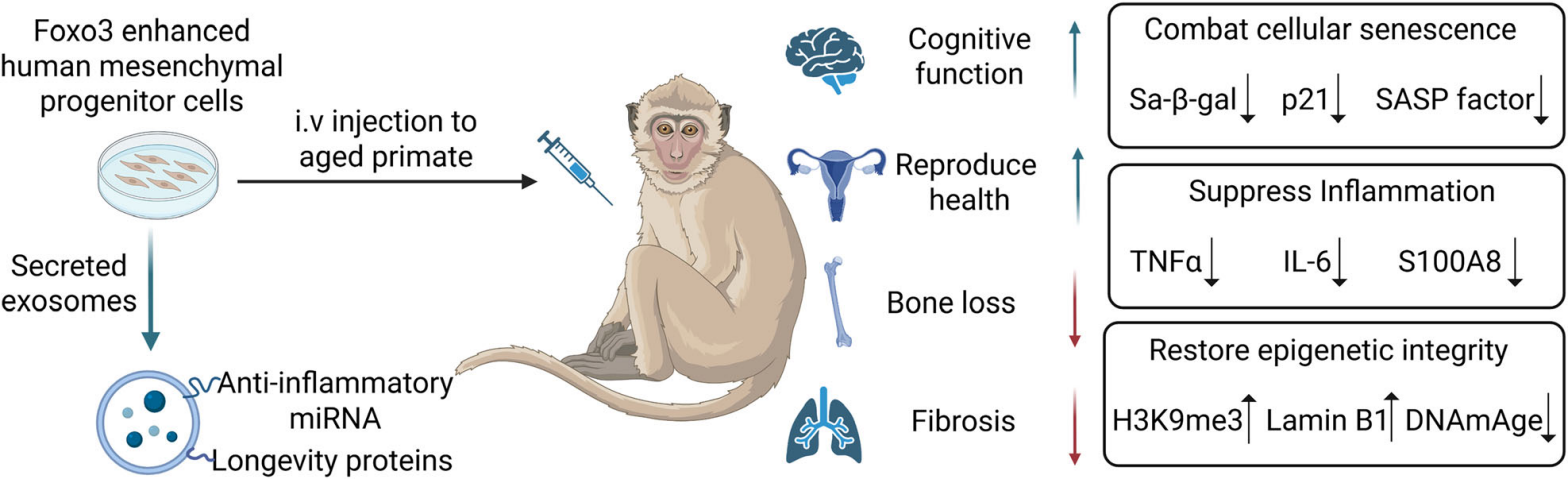
Administration of senescence-resistant human mesenchymal progenitor cells induce multi-organ rejuvenation in aging primates. Senescence-resistant human mesenchymal progenitor cells (SRCs) were engineered by constitutive activation of FOXO3. These SRCs was intravenously administrated to aged macaques in a 44-week study. SRC treatment systemically ameliorated age-related pathologies, including cognitive decline, osteoporosis, fibrosis, and infertility. SRC-mediated rejuvenation was partially attributed to SRC-derived exosomes, which possess the ability to counteract cellular senescence, suppress inflammation, and restore epigenetic integrity.

## The science of defying time

Aging is not a single blow but a slow-motion collapse at the systemic and cellular level. Stem cells, the body’s repair crews, lose their regenerative edge over time, leaving tissues vulnerable to decay [4]. This triggers a vicious cycle: chronic inflammation festers, cellular senescence spreads, and age-related diseases take hold [5]. Past interventions—caloric restriction, metformin, or rapamycin—have shown promise in worms or mice but faltered in primates [6]. Human MPCs, however, have long been investigated for their anti-aging effects [7]. Used for treating conditions like arthritis and premature ovarian failure, MPCs boast low immunogenicity, making them potentially useful also for cross-species (xenogeneic) therapies, and a knack for quelling inflammation, a key driver of aging [8, 9].

Yet, the aging body is a brutal environment, where oxidative stress and systemic inflammation can sabotage transplanted cells. According to Liu’s team, SRCs are MPCs genetically enhanced to resist senescence, the state where cells stop dividing and spew toxic signals that accelerate aging. By integrating FOXO3, a gene linked to longevity, researchers created cells that not only endure but actively counteract aging. This builds on recent findings of longevity pathways, requiring minimal genetic tweaks for maximum impact. While earlier studies tested MPCs for localized injuries in rodents, this is the first to explore SRCs’ systemic anti-aging effects in primates—a critical bridge to human relevance.

## A primate revolution

Researchers compared FOXO3-enhanced SRCs to wild-type MPCs, in a classic 44-week macaque trial. SRCs didn’t just outperform; they seem to rewrite the aging playbook. Biological aging clocks—markers of cellular and tissue decline—ticked backward. Chronic inflammation, a hallmark of aging, as well as cellular senescence, the scourge of vitality, were stably significantly reduced. Not only the macaques didn’t merely age more slowly; they showed signs of rejuvenation across multiple systems.

SRC treatment bolstered neural connectivity and cognitive function. The skeletal system gained density. The reproductive system seemed to show restored vitality. Now the question is on the distinct tissue-specific targets of the exosomes.

## The path forward

Cynomolgus macaques are close cousins to humans, but translating SRCs to clinical trials requires navigating a gauntlet of challenges. Will SRCs perform as well in humans, with our diverse genetics and lifestyles? How durable are the benefits? And what are the societal costs of extending lifespan—could it deepen inequalities? These questions open a new venue of research. The macaque results are a proof of concept, a tantalizing vision of what’s possible. Aging populations are straining global healthcare systems, with age-related diseases costing trillions annually. SRCs, by targeting aging’s root elicit new future studies and translating in to human geriatric medicine. Although Lei et al. describe clear effect decelerating aging in non-human primates, further research is warranted in modulating systemic homeostasis, exploring mechanisms beyond exosomes.

A key question is how aging stromal progenitors lose rejuvenation capacity and what molecular components, replaced by SRCs, are deficient. SRCs likely secrete bioactive factors—cytokines, growth factors, and exosomes—mimicking rejuvenating signals from young partners as seen in parabiosis. Identifying these, such as klotho, sirtuins, or miR-146a, could decode rejuvenation mechanisms. Future research should profile SRC-derived factors using multi-omics, investigate epigenetic and microenvironmental causes of progenitor decline, develop scalable exosome-based therapies targeting inflammaging and age-related diseases like Alzheimer’s, and explore personalized therapies using patient-specific data. These efforts will illuminate rejuvenation forces, driving innovative interventions for healthy aging and age-related pathologies.
